# A Critical Point of Male Gonad Development: Neuroendocrine Correlates of Accelerated Testicular Growth in Rats during Early Life

**DOI:** 10.1371/journal.pone.0093007

**Published:** 2014-04-02

**Authors:** Nikolay N. Dygalo, Tatjana V. Shemenkova, Tatjana S. Kalinina, Galina T. Shishkina

**Affiliations:** 1 Institute of Cytology and Genetics Russian Academy of Sciences, Laboratory of Functional Neurogenomics, Novosibirsk, Russia; 2 Novosibirsk State University, Department of Physiology, Novosibirsk, Russia; University of Rouen, France, France

## Abstract

Testis growth during early life is important for future male fertility and shows acceleration during the first months of life in humans. This acceleration coincides with the peak in gonadotropic hormones in the blood, while the role of hypothalamic factors remains vague. Using neonatal rats to assess this issue, we found that day 9 of life is likely critical for testis development in rats. Before this day, testicular growth was proportional to body weight gain, but after that the testes showed accelerated growth. Hypothalamic kisspeptin and its receptor mRNA levels begin to elevate 2 days later, at day 11. A significant increase in the mRNA levels for gonadotropin-releasing hormone (GnRH) receptors in the hypothalamus between days 5 and 7 was followed by a 3-fold decrease in GnRH mRNA levels in this brain region during the next 2 days. Starting from day 9, hypothalamic GnRH mRNA levels increased significantly and positively correlated with accelerated testicular growth. Triptorelin, an agonist of GnRH, at a dose that had no effect on testicular growth during “proportional” period, increased testis weights during the period of accelerated growth. The insensitivity of testicular growth to GnRH during “proportional” period was supported by inability of a 2.5-fold siRNA knockdown of GnRH expression in the hypothalamus of the 7-day-old animals to produce any effect on their testis weights. GnRH receptor blockade with cetrorelix was also without effect on testis weights during “proportional” period but the same doses of this GnRH antagonist significantly inhibited “accelerated” testicular growth. GnRH receptor mRNA levels in the pituitary as well as plasma LH concentrations were higher during “accelerated” period of testicular growth than during “proportional” period. In general, our data defined two distinct periods in rat testicular development that are primarily characterized by different responses to GnRH signaling.

## Introduction

Testis growth during early life is important for future male fertility. Testicular development depends on intrinsic and environmental factors in man [1]–[3] and rodents [4]. In humans, there is a period of accelerated testicular growth from birth to 5 months of age [5]. This significant rise in testicular volume coincides with the peak in gonadotropic hormones during the first months of age [6]–[9]. The data on changes in expression of hypothalamic factors, such as gonadotropin-releasing hormone (GnRH) and kisspeptin, during the accelerated phase of the normal testicular growth remain incomplete due in part to the absence of relevant experimental model. Even though there are species differences in testicular development, the main stages of this process may be similar in all mammals [10], [11]. Thus, some signs of testicular growth acceleration may be expected in rat pups. If so, rodents could be used for investigation of hypothalamic neuroendocrine correlates of testicular growth in neonatal mammals.

Kisspeptin, GnRH and their receptors that regulate pituitary luteinizing hormone (LH) and follicle-stimulating hormone (FSH) secretions are key players in the hypothalamic mechanisms of the onset of puberty [12]. All these factors are already expressed in both rat hypothalamus and pituitary at birth [13], [14]. However, their role in the development of gonads and gonadal steroid function during perinatal period remains a matter of debate [15]–[17]. Thus, despite the well-known ability of exogenous gonadotropins as well as GnRH agonists to stimulate testicular growth still in prenatal rats [18]–[20], no association was observed between the development and functioning of the rodent testes and hypothalamic GnRH as well as GnRH targets, LH and FSH, in the pituitary during intrauterine period [16]. Moreover, perinatal peak of testosterone secretion that is important for sexual differentiation of the brain was shown to be independent of GnRH and kisspeptin [21]. Plasma testosterone levels that peaked at the perinatal period thereafter decline and remain low until the third week of life [22], [23].

In contrast to testosterone, testis weight is increased progressively day by day during neonatal period. Testicular growth was inhibited in neonatal mice lacking GnRH [24]. Proliferation of the main type of testicular somatic Sertoli cells is active in rodents during the first two weeks of life and ceased by the day 15 [25]. In these animals, testicular testosterone producing Leydig cells of adult LH-dependent type begin to substitute the cells of fetal type after the first week of life [16]. The data on mice deficient in kisspeptin or GnRH signaling provide evidence that hypothalamus begins to have a significant impact on rodent testis development somewhere within the first 2 weeks of life [16], [21]. However, functional significance of hypothalamic factors for testis growth during early postnatal period in genetically normal animals remains unclear. To investigate these issues, we analyzed the growth of the testes and mRNA levels for GnRH, kisspeptin and their receptors in the preoptic area of the hypothalamus of rat pups during the first two weeks of life. In addition, the effects of siRNA inhibition of GnRH expression and treatment with GnRH agonist and antagonist on testicular growth in 1- and 2-week-old rats were also assessed.

## Experimental Procedures

### Animals

All animal use procedures in this study were supervised and specifically approved by the ethics committee of the Institute of Cytology and Genetics Russian Academy of Sciences in compliance with the International European ethical standards (86/609-EEC) and the Russian national instructions for the care and use of laboratory animals (N 267 19.06.2003). All efforts were made to minimize animal suffering.

Wistar rat pups were used in this study. The dams had free access to food and water. On postnatal day 2, litters were culled to 8 pups, and only males were used in experiments. Rats were weighted and sacrificed by decapitation at the ages of 3, 5, 7, 9, 11 and 14 days. Testes were dissected and weighted. The brains were removed from the skull and anterior hypothalamic preoptic area was dissected from the brain. The caudal boundary of the anterior hypothalamic preoptic area was at the middle of the tuber cinereum. For the sake of comparison, this area was also dissected from the brains of adult male rats. Blood samples and pituitaries were collected at 7 and 14 days of age. All tissue samples were stored in liquid nitrogen until further processing.

### siRNA knockdown of GnRH expression

In knockdown experiment, pups were injected with short interfering RNA (siRNA) targeting GnRH mRNA from 369 to 389 (sense — 5′-uacagagcuguaacaggucat -3′ and antisense — 5′- gaccuguuacagcucuguaat -3′strands; 200 μM in 5 μl of OptiMEM, Invitrogen, United States) into the anterior hypothalamic preoptic area (5 mm rostral of lambda and 5 mm lower than the scull) on day 5 of life under cold anesthesia [26]. It was shown previously that direct injection of selective siRNA into neonatal rat brain effectively blocks targeted gene expression [27], [28]. Animals of control groups received the same volume of vehicle or the same amount of control short double stranded RNA (dsRNA; the first — 5′- aagucucguauguagugguu-3′ and the second — 5′-ccacuacauacgagacuuguu-3′ strands), which has no appreciable homology with any rat mRNA. The oligonucleotides were chemically synthesized, purified and annealed for duplex RNA formation. Animals from control and experimental groups were represented in each litter. Rats of these groups were sacrificed at day 7 of life.

### Treatment with GnRH agonist triptorelin and antagonist cetrorelix

Triptorelin (0.1 mg/kg), a synthetic analog of GnRH (Ipsen Pharma Biotech, France), or cetrorelix (0.1 and 0.5 mg/kg), a GnRH antagonist (ASTA Medica AG, Germany), were injected subcutaneously (in 25 μl of saline/day) for 3 consecutive days (5, 6, 7 or 12, 13, 14 days of life). Animals of appropriate control groups received the same volume of saline or remained without any injections. Six hours after the last injection, all these pups at ages of 7 or 14 days, respectively, were sacrificed, and their body and testicular weights were determined.

### Analysis of mRNAs for GnRH, kisspeptin and their receptors, FSH-β, LH-β and beta-actin by Real-time PCR

Total cellular RNA was isolated by a single step acidic phenol extraction. Only the RNA samples with 260/280 ratios of 1.8–2.0 were used for subsequent analyses. First strand cDNA was synthesized from 5 μg of each RNA sample with 500 nM Oligo(dT) primer and 50 U MuLV reverse transcriptase (SibEnzyme, Russia) in 20 μl of reaction mixture containing 50 mM Tris–HCl (pH 8.3), 75 mM KCl, 5 mM MgCl2, 0.01 M DTT, 1 mM dNTP mix at 42°C for 1.5 h.

The TaqMan assay-based real-time PCR was performed using TaqMan Gene Expression Assays (GnRH - Rn00562754_m1; GnRHR - Rn01413644_m1; Kiss1 - Rn00710914_m1; Kiss1R - Rn00576940_m1; FSH-β - Rn01484594_m1; LH-β - Rn00563443_g1; beta-actin - Rn01424440_s1; Applied Biosystems, Foster City, CA) and the ABI Prism 7000 Sequence Detection system (Applied Biosystems) as was described previously [17]. All reactions were carried out in triplicate on cDNA samples in 96-well optical plates according to the manufacturer's protocol in 25 μl of 1 × TaqMan Universal PCR Master Mix (Applied Biosystems). The real-time PCR consisted of one cycle of 50°C for 2 min and 95°C for 10 min, followed by 40 cycles each of 95 oC for 15 s and 60°C for 1 min. The comparative ddCT method was used to calculate mRNA expression relative to the beta-actin as an endogenous control according to manufacturer's manual (Applied Biosystems). There were no age-related differences in beta-actin mRNA levels and this housekeeping gene was used to normalize mRNA levels of the genes of interest.

### Plasma LH and testosterone measurement

ELISA kits were used for determination of testosterone (sensitivity 0.05 ng/ml, intra assay variation 8%, range of evaluated concentrations 0–18 ng/ml) and LH (sensitivity 0.25 mIU/ml, intra assay variation 8%, range of evaluated concentrations 0–100 mIU/ml) levels in the blood plasma (Alkor Bio, St. Petersburg, Russia). Trunk blood was collected from 7 and 14 day-old rats into cold tubes containing 0.05 ml of 0.25M EDTA, centrifuged at 4°C for 20 min, and then the plasma was stored at −60°C until assay.

### Statistical analysis

Data were evaluated by one-way ANOVA, linear regression and Pearson's correlation analysis using SYSTAT statistical software (SYSTAT 6.0, Chicago, IL). One-way ANOVA followed by Tukey's post hoc test was used to compare (1) body weights and absolute testes weights as well as expression of each gene between the different postnatal days and (2) GnRH mRNA levels as well as relative testis weights between treatment groups in experiments with siRNA knockdown, triptorelin and cetrorelix. Mean differences between groups with P less 0.05 were considered statistically significant. Data are expressed as mean ± SEM.

## Results

### Testicular growth

**Figure 1:**
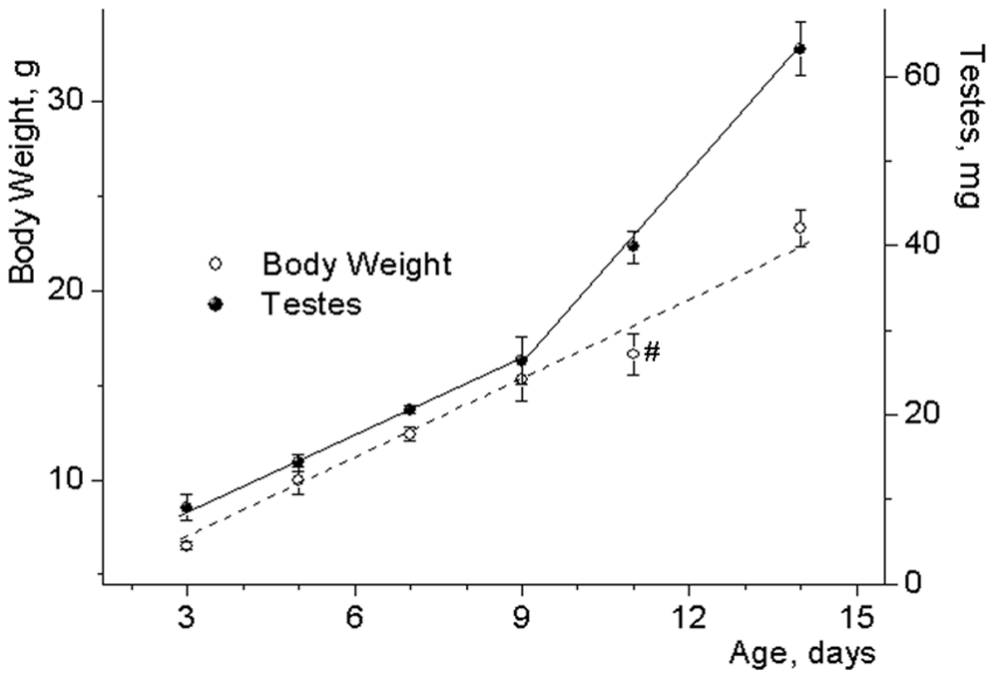
Testicular growth in neonatal rats. Male rats were weighted and sacrificed at the ages of 3, 5, 7, 9, 11 and 14 days. Testes were dissected and weighted. Data represent mean ± SEM of 6–8 individual animals. Regression lines for testis weights (TW): {[TW (days 3–9, mg)]  = 0.043+2.923 [Day]} and {[TW (days 9–14, mg)]  = −40.782+7.407 [Day]}. The difference between linear slope coefficients modeling the rate of change in testes weight before (b1 = 2.923±0.062, n = 4) and after day 9 (b2 = 7.40716±0.299, n = 3) was significant (p<0.001). All means were significantly different (p<0.05) from younger and older groups except the mean body weight of 11-day-old pups (#) that did not differ from the 9-day-old group as analyzed by one-way ANOVA followed by Tukey's post hoc test.

During the first 2 weeks of postnatal development, body weights increased approximately 3.5-fold [F(5,37)  = 34.298, p<0.001] and absolute paired weights of the testes increased 7-fold [F(5,37)  = 39.336, p<0.001) (Figure 1).

Before day 9, body and testis weights increased in parallel. Testis growth between days 3 and 9 can be described by linear regression (Equation 1). The regression coefficient is highly significant (r = 0.999, p<0.001). The relative weights of the testes did not change (F2, 17 = 0.504, p = 0.613) for the first week of life.
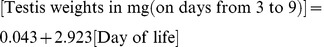
(Equation 1)


Testis growth rate begins to exceed body weight gain after day 9. Rate of testicular growth after day 9 satisfies the equation (2). The regression coefficient for the Equation 2 is also highly significant (r = 0.999, p<0.05). In contrast to the first week, the relative weights of testes significantly increased with age after day 9 [F(2, 21)  = 18.533, p<0.001].
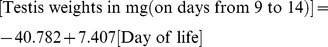
(Equation 2)


The difference in the linear slope coefficients modeling the rate of change in testes weight before (b1 = 2.923±0.062, n = 4) and after (b2 = 7.40716±0.299, n = 3) day 9 was significant (p<0.001). Thus, testicular growth is qualitatively different before and after day 9 of life. It passively follows the general growth rate of the animal during the first period - before day 9 (period of proportional growth), and after this day, during the second period, male gonads undergo accelerated growth.

### Expression of GnRH, kisspeptin and their receptors in the preoptic area of the hypothalamus

**Figure 2:**
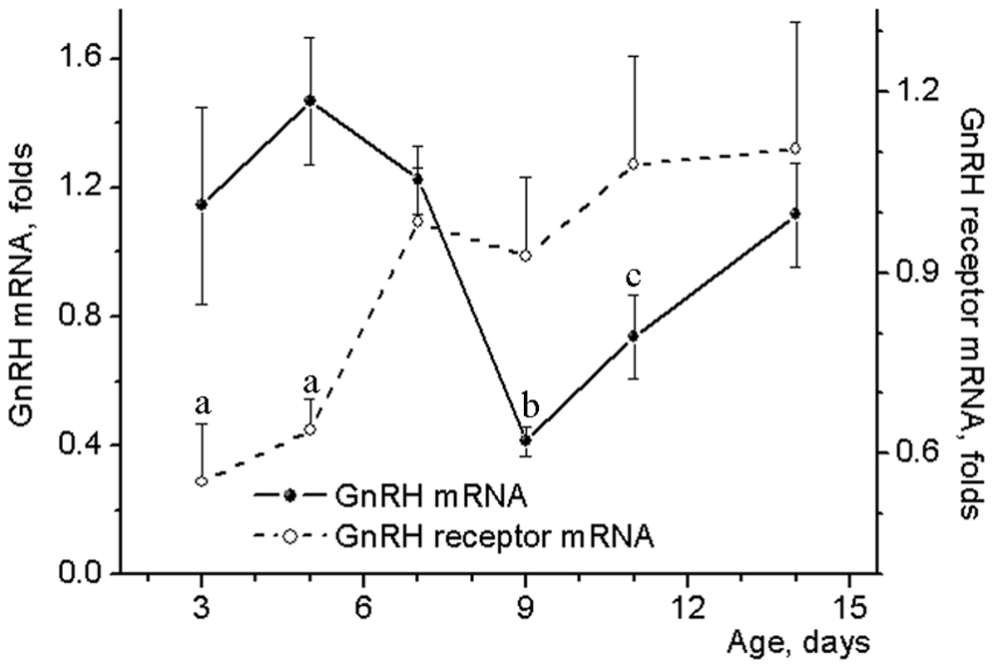
GnRH and its receptor mRNA levels in the hypothalamus of neonatal male rats. Probes of cDNA were synthesized from total mRNA extracted from the anterior hypothalamic preoptic area of 3–14 day-old male rats. Probes were examined for GnRH and its receptor mRNA expression by the TaqMan assay-based real-time PCR. The comparative ddCT method was used to calculate mRNA expression relative to the beta-actin as an endogenous control. Data represent mean ± SEM of 6–8 individual animals. **^a^**p<0.05 compared with 7-14-day old groups, **^b^**p<0.05 compared with 3–7- and 14-day old groups, **^c^**p<0.05 compared with 5-7-day old groups as analyzed by one-way ANOVA followed by Tukey's post hoc test.

GnRH mRNA levels in the hypothalamus of 3-7-day-old male rats (Figure 2) did not differ from that (1.22 ± 0.18 folds to beta actin mRNA) in adult rats. Hypothalamic GnRH mRNA expression then decreased (3-fold) between days 7 and 9 and returned to initial values during the next 5 days [F(5,35)  = 3.076, p<0.05]. The transient decrease in the GnRH mRNA levels was preceded by a marked 2-fold increase in the GnRH receptor gene expression between days 5 and 7 [F(5,35)  = 3.200, p<0.01] (Figure 2). Thereafter, this receptor mRNA levels remained high and did not change significantly from days 7 to 14. In 14-day-old animals, GnRH receptor mRNA levels were almost 2 times higher than that (0.58 ± 0.10 folds to beta actin mRNA) in adult males.

**Figure 3:**
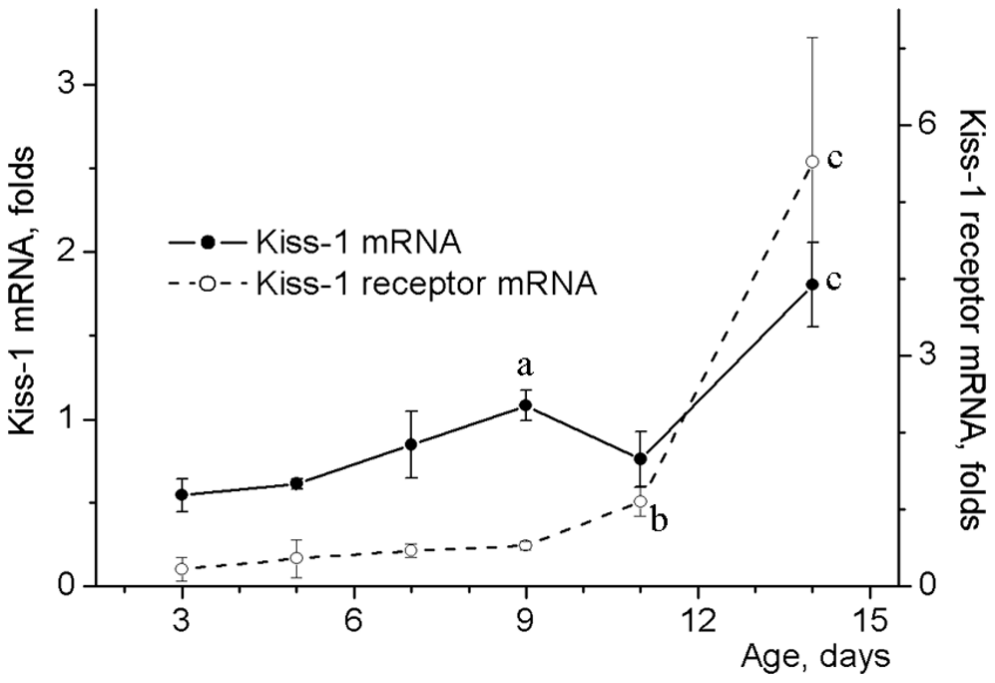
Kisspeptin and its receptor mRNA levels in the hypothalamus of neonatal male rats. Probes of cDNA were synthesized from total mRNA extracted from the anterior hypothalamic preoptic area of 3–14 day-old male rats. Probes were examined for kisspeptin and its receptor mRNA expression by the TaqMan assay-based real-time PCR. The comparative ddCT method was used to calculate mRNA expression relative to the beta-actin as an endogenous control. Data represent mean ± SEM of 3–6 individual animals. **^a^**p<0.05 compared with 3-5-day old groups, **^b^**p<0.05 compared with 3-9-day old groups, **^c^**p<0.05 compared with 3-11-day old groups as analyzed by one-way ANOVA followed by Tukey's post hoc test.

Kisspeptin and its receptor mRNA levels in the hypothalamus of 3 day-old rats were more than 10 and 6 times lower than that (kisspeptin: 5.92 ± 1.51 folds; receptors: 1.36 ± 0.12 folds to beta actin mRNA) in adult males, respectively (Figure 3). Kisspeptin expression increased 2-fold between days 3 and 9 and reached 30% of adult male levels at day 14 [F(5,24)  = 4.299, p<0.01]. Similar to kisspeptin, its receptor mRNA levels also doubled from day 3 to day 9 and increased further to almost 4-fold of the level of adult male at day 14 [F(5,13)  = 4.490, p<0.05].

### Neuroendocrine correlates of testis growth in the neonatal rat

**Figure 4:**
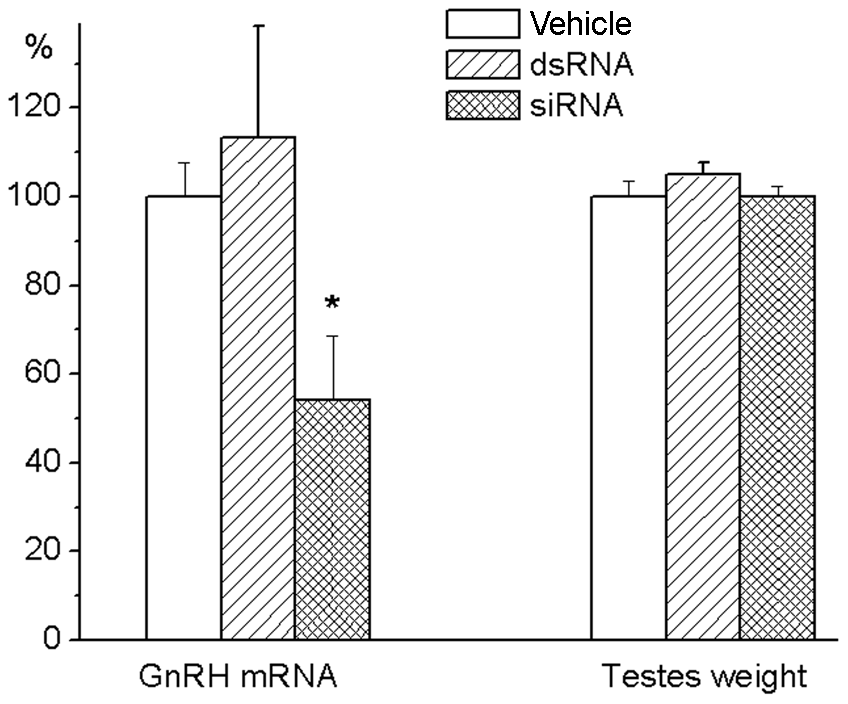
Testes weight in 7-day old rats after siRNA knockdown of GnRH expression. siRNA targeting GnRH mRNA from 369 to 389 (200 μM in 5 μl of OptiMEM) was injected into the anterior hypothalamic preoptic area on the day 5 of life. Animals of control groups received the same volume of vehicle or the same amount of control short double stranded RNA (dsRNA), which has no homology with any rat mRNA. Rats were weighted and sacrificed at the day 7 of life. Testes were dissected and weighted. Probes of cDNA were synthesized from total mRNA extracted from the anterior hypothalamic preoptic area. Probes were examined for GnRH mRNA expression by the TaqMan assay-based real-time PCR. The comparative ddCT method was used to calculate mRNA expression relative to the beta-actin as an endogenous control. Data are expressed as percentages of the values for the vehicle-treated control group that was taken as 100%, and presented as mean ± SEM of 7 individual animals. *p<0.05 compared with vehicle and dsRNA groups as analyzed by one-way ANOVA followed by Tukey's post hoc test.

There were no significant interrelations between testis weights and hypothalamic mRNA levels for kisspeptin or its receptors during both proportional and accelerated periods of testis growth. There were also no significant relationship between the levels of GnRH mRNA in the hypothalamus and the relative weights of the testes in 3–7 day-old rats. The independence of the growth rate of testes from GnRH during this period of development was supported by experiments in which GnRH expression was suppressed by RNA interference (Figure 4). A significant decrease in the level of GnRH mRNA [F(2,19)  = 3.885, p<0.05] in the hypothalamus of 7-day-old rat pups after administration of siRNA into their brain on day 5 had no effect on the weights of the testes [F(2,19)  = 0.530, NS].

**Figure 5:**
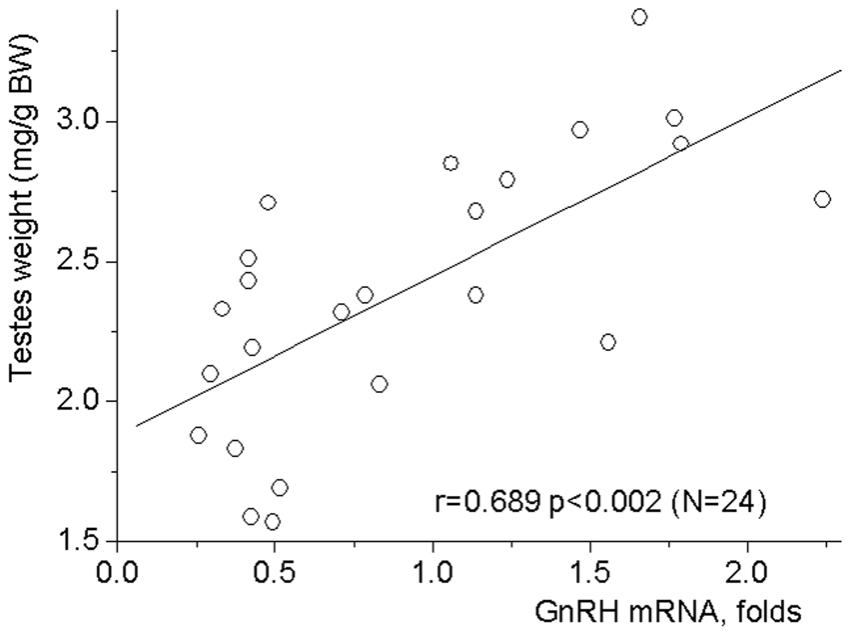
Correlation between testes weights and GnRH mRNA levels in the hypothalamus. Rats were weighted and sacrificed at the days 9, 11 and 14 of life. Testes were dissected and weighted. Probes of cDNA were synthesized from total mRNA extracted from the anterior hypothalamic preoptic area. Probes were examined for GnRH mRNA expression by the TaqMan assay-based real-time PCR. The comparative ddCT method was used to calculate mRNA expression relative to the beta-actin as an endogenous control. Each point represents an individual animal. Significant Pearson's linear correlation (r = 0.689 p<0.005, N = 24) was found between testes weights and GnRH mRNA levels.

**Figure 6:**
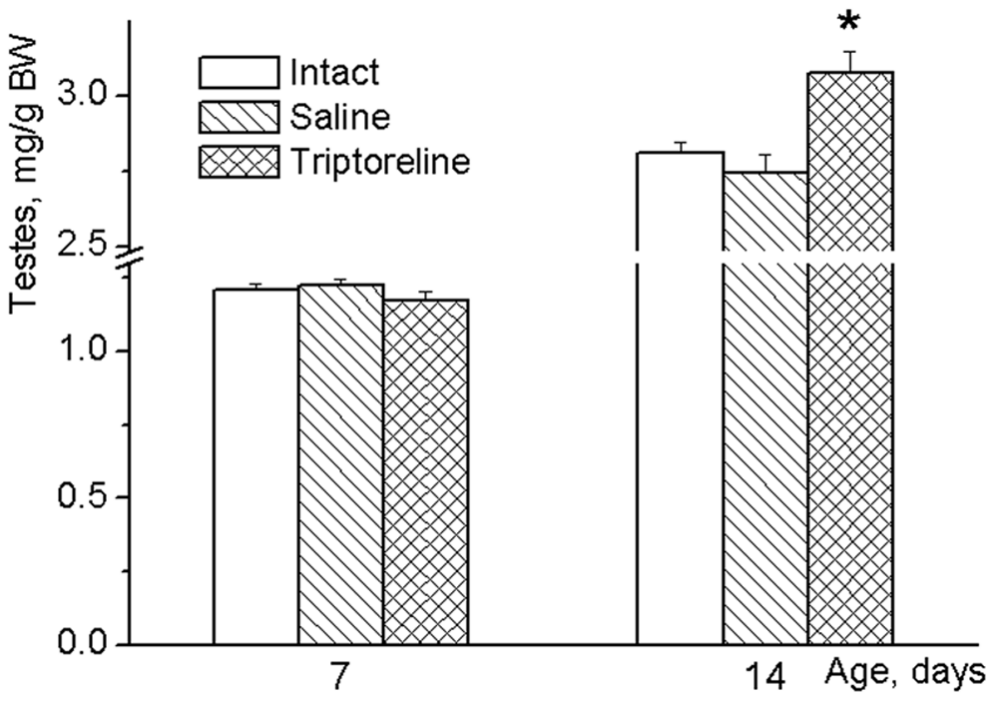
Testes weights of 7 and 14 day-old rats after treatment with GnRH agonist triptorelin. Triptorelin was injected subcutaneously (100 μ/kg in 25 μl of saline/day) for 3 consecutive days (5, 6, 7 or 12, 13, 14 days of life). Animals of control groups received the same volume of saline or remained without any injections. Six hours after the last injection, all these pups at ages of 7 or 14 days, respectively, were sacrificed, and their body and testicular weights were determined. Data are presented as mean ± SEM of 9–10 individual animals. *p<0.05 vs. intact and saline-treated groups as analyzed by one-way ANOVA followed by Tukey's post hoc test.

In contrast to the period from day 3 to day 7, a highly significant positive correlation (r = 0.689, p<0.01, N = 24) between the relative weights of the testes and the hypothalamic GnRH mRNA levels was found on days 9–14 of life (Figure 5). The sensitivity of testes to GnRH signal was higher during the second as compared to the first week of life. Thus, treatment with a synthetic GnRH agonist triptorelin for three days that had no effect on the weights of the testes in 7-day-old rats [F(2,50)  = 0.600, NS], increased the weights of the gonads at day 14 [F(2, 53)  = 7.149, p<0.01] (Figure 6). These data indicate an increase in sensitivity of testicular growth to exogenous stimulation with GnRH analog during the second week of life.

**Figure 7:**
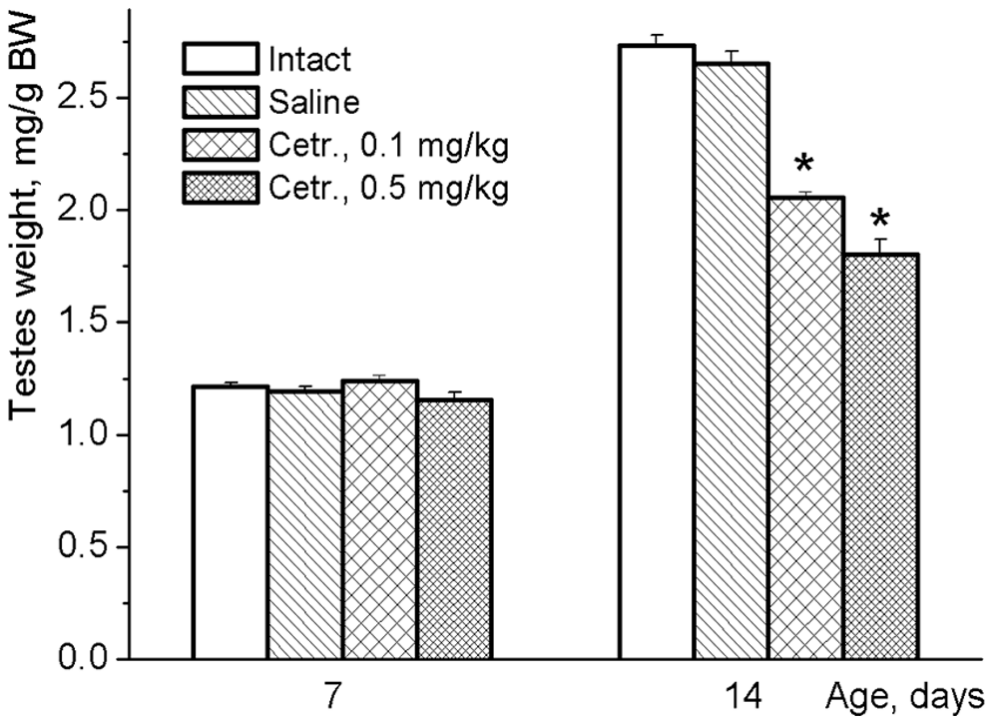
Testes weights of 7 and 14 day-old rats after treatment with GnRH antagonist cetrorelix. Cetrorelix was injected subcutaneously (0.1 or 0.5 mg/kg in 25 μl of saline/day) for 3 consecutive days (5, 6, 7 or 12, 13, 14 days of life). Animals of control groups received the same volume of saline or remained without any injections. Six hours after the last injection, all these pups at ages of 7 or 14 days, respectively, were sacrificed, and their body and testicular weights were determined. Data are presented as mean ± SEM of 9–10 individual animals. *p<0.001 vs. intact and saline-treated groups as analyzed by one-way ANOVA followed by Tukey's post hoc test.

The impact of endogenous GnRH signaling on testes was much stronger during the period of their accelerated growth than in the period of proportional growth (Figure 7). Pharmacological blockade of GnRH receptors with increasing doses of GnRH antagonist cetrorelix that had no appreciable effects on testes in a week-old rats [F(3, 55)  = 0.909, NS], significantly and dose-dependently inhibited testicular growth in 2-week-old animals [F(3, 63)  = 31.866, p<0.001].

In addition to specific interrelations between testis weight and GnRH signaling, periods of proportional and active testicular growth are characterized by important differences in pituitary gene expression (Table 1) and hormone levels in the blood (Table 2). There was a significant increase of GnRH receptor mRNA levels in the pituitary between the first and second week of life. While pituitary FSH-β mRNA levels did not change, unexpectedly, LH-β mRNA levels significantly decreased during this period. LH levels in the blood did not follow changes in LH-β mRNA expression and increased significantly between days 7 and 14. Plasma testosterone levels were similar in one- and two-week-old rats.

**Table 1:** Levels of mRNA for GnRH receptor, FSH-β and LH-β in the pituitary of neonatal male rats.

	7-day-old	14-day-old
GnRH receptor	1.05±0.07 (14)#	1.60±0.15 (15)*
FSH-β	1.19±0.12 (12)	1.26±0.15 (13)
LH-β	1.56±0.15 (13)	0.69±0.05 (15)*

# mean ± SEM (N), folds to β-actin mRNA; N – number of probes. *p<0.05 in comparison with day 7, Tukey's test.

**Table 2:** Plasma LH and testosterone levels in neonatal male rats.

	7-day-old	14-day-old
LH (mIU/ml)	1.17±0.05 (22) #	1.60±0.20 (21)*
Testosterone (ng/ml)	0.98±0.10 (22)	1.06±0.11 (20)

# mean ± SEM (N), N – number of probes. *p<0.05 in comparison with day 7, Tukey's test.

## Discussion

We have shown that testicular growth undergoes acceleration in rats after day 9 of life. Similar acceleration of testicular growth in rodents after the first postnatal week could be found in other publications (for example, Figure 5 in Kerr and Knell, 1988 [29]; Figure 5 in Barakat et al., 2008 [30]; Figure 1 in Zhang et al., 2004 [31]; Table 1 in Buzzard et al., 2004 [32]) but has never been described and discussed previously.

Sertoli cells are the main cell type in the testis, the number of which correlates well with the size of the testis [33]. The ability of these cells to respond to FSH was significantly augmented on day 11 as compared to the responses in 9-day-old rats [34]. This switch in Sertoli cells from FSH resistant to FSH responsive mode may contribute to acceleration of testicular growth found in the present study.

At the pituitary level, there were no differences in FSH-β mRNA levels between the periods of proportional and accelerated testicular growth. This is in line with data showing that in male rats, serum FSH remains at relatively low levels for 3 weeks [35]–[37]. Some decrease in pituitary LH-β mRNA levels between days 7 and 14 may result from maturation of feedback control of gonadotropin subunit gene expression. Pituitary LH-β mRNA expression appears to be more sensitive compared to FSH-β mRNA to the feedback effect of sex steroids as was shown in adult male mice [38] and in neonatal female rats [39]. Diethylstilbestrol treatment significantly reduced pituitary LH and FSH expression at days 10 and 15, but only LH-β mRNA levels were significantly increased after gonadectomy in 15-day-old animals, but not in 10-day-old animals [39]. Normal feedback down-regulation of LH-β mRNA expression requires aromatization of testosterone and activation of estrogen receptor signaling pathways in the gonadotrope [38]. While there were no changes in testosterone levels in the blood, in the rat pituitary, estrogen receptor number increased 2-fold between postnatal days 7–8 and 10–11 [40], and this might facilitate feedback regulation of the gonadotrope. The disparity in age related changes in levels of LH-β mRNA and plasma LH concentrations could be explained by that most regulation of plasma LH concentrations occurs at the level of secretion, rather than subunit gene expression [41]. The increase in LH concentrations in the blood of 2-week-old male rats compared to 7-day-old animals was accompanied by an increase in expression of the pituitary GnRH receptor that mediates stimulation of gonadotropes by the hypothalamic GnRH.

Testis growth is sensitive to exogenous gonadotropins and GnRH agonists in prenatal rats [18]-[20]. The responses of LH and GnRH could be elicited by exogenous kisspeptin in 5-day-old rats [42]. However, a significant retardation of the testis growth was found in rodents genetically deficient in hypothalamic GnRH, kisspeptin as well as pituitary FSH or LH signaling approximately at days 5–10 [16], [21], [31]. In our experiments, expression of kisspeptin, an important regulator of sexual development, as well as its receptor did not increase significantly within this time frame and increased only after day 11 of life - two days later after the beginning of testicular growth acceleration. The early study using semi-quantitative RT-PCR and SYBR Green real-time RT-PCR did not reveal any significant alterations in kisspeptin or its receptor mRNA levels in the rat hypothalamus determined at the 1, 5, 10 and 15 days of life [43]. Using more sensitive method, Walker et al. [44] recently found an increase in kisspeptin mRNA levels in the rat hypothalamic regions between postnatal days 5 and 15. The developmental profile of kisspeptin mRNA obtained in our study with real-time PCR is in agreement with previous data [44] and well coincides with immunohistochemical identification of kisspeptin expression. During the first 2 weeks of life, low-level immunoreactivity for kiss-1 was detected in the male rat arcuate nucleus [45]. Though mice lacking kisspeptin-Kiss1r signaling had smaller testes already at postnatal day 10 [21], kisspeptin or its receptor mRNA levels in the present study did not correlate with testis weights during the periods of the proportional and accelerated growth of the gonads. It seems that kisspeptin does not have an obvious function in neonatal acceleration of testicular growth. This suggests involvement of kisspeptin-independent regulators in this process. The best candidate for this role appears to be GnRH because it retains some degree of biological activity in knockout mice lacking genes for kisspeptin or its receptor [46].

Unlike expression of genes related to kisspeptin signaling, mRNA levels for GnRH and its receptor undergo significant changes within short time intervals before and during acceleration of testicular growth. These alterations in the expression of genes involved in GnRH signaling lasted only a few days, and thus may have been missed in previous studies due to longer intervals between sampling that were usually 5–10 and even more days [44], [47]–[49]. The most increase in the GnRH receptor mRNA levels was found between days 5 and 7. The activation of the receptor expression was followed by a 3-fold decrease in mRNA levels for GnRH during the next two days. Then, GnRH mRNA levels restored stepwise to values that were in a week-old animals. GnRH mRNA levels were well correlated with testis weights after day 9. However, a stepwise increase in this mRNA levels is an unlikely proximate cause of accelerated testicular growth. This acceleration begins at a relatively low GnRH mRNA levels. Moreover, while testicular growth between days 9 and 11 as well as between days 11 and 14 was highly significant, the levels of GnRH mRNA did not change significantly within both periods. Thus, the changes in the levels of GnRH and its receptor mRNA may be indicators of GnRH neuron development and function, rather than a direct cause of accelerated testicular growth.

The changes in GnRH and its receptor mRNA levels may be interrelated. GnRH neurons and, for example, hypothalamic GABA-neurons express receptors for GnRH [50]. These receptors are involved in direct [51] and indirect [50] regulation of GnRH-neuron activity as well as in the release of GnRH, and potentially could affect GnRH gene expression [52]. The developmental pattern of GnRH mRNA found in the present study may be related to the dependence of GnRH neuron activity on GnRH feedback signal. Low levels of GnRH inhibit activity of the GnRH neuron whereas the high levels of the peptide stimulate the neuron directly [53] and/or indirectly [50]. Before the increase in expression of GnRH receptors between days 5 and 7, there was no significant GnRH input to GnRH neuron. GnRH receptors expression reached high level at day 7, but unsynchronized at this age secretion of GnRH by individual neurons was able to produce only weak GnRH signal to GnRH neurons and thus temporally inhibited their activity. The decrease in GnRH mRNA levels found between days 7 and 9 suggests this inhibition. Synchronization of GnRH secretion by remote from one another neurons presumably occurred after day 9. A morphological basis for this synchronization may be dendro-dendritic bundling and shared synapses found between GnRH neurons [54]. In rodents, at postnatal day 3, most GnRH neurons appeared as a simple oval immunoreactive soma and at postnatal day 10 these neurons exhibit a highly branched/complex dendritic tree in the rostral preoptic area [55]. GnRH pulses generated by synchronized release of the peptide in conjunction with a high level of the receptor expression were capable of enhancing GnRH signaling to GnRH neurons and thus activate these neurons as well as GnRH gene expression found in our study after day 9. The acceleration of testicular growth suggested that GnRH pulses generated at this age might begin to have a significant stimulating effect on rat testis in vivo. This interpretation is supported by experiments with GnRH neurons of mouse fetal nasal explants showing that amplitude of GnRH pulses and the secretory rate gradually increased with culture days, reaching the maximum at 2–3 weeks [56]. A pronounced increase in endogenous GnRH input to the pituitary which in turn stimulates testicular growth at the middle of the second week of life was clearly demonstrated by experiments in which GnRH signaling was modulated pharmacologically. Even the high dose of GnRH antagonist cetrorelix as well as siRNA-mediated knockdown of GnRH mRNA and GnRH agonist triptorelin had no effects on testes during “proportional” period of their growth. In contrast, during “accelerated” period, GnRH antagonist significantly decreased, whereas its agonist increased testicular weight. It appeared that testis growth was relatively insensitive to both endogenous and exogenous GnRH signals during “proportional” period, while during “accelerated” period, susceptibility of testes to these signals was high.

In conclusion, by a detailed study of neuroendocrine correlates of testis growth in neonatal rats, we defined for the first time two distinct periods of testicular development. In the earlier period, up to day 9 of life, testicular growth is proportional to the body weight gain and after this day, during the second period, the testes undergo accelerated growth. These periods are primarily characterized by different dependence of testicular development on GnRH signaling. Testes are relatively insensitive to alterations in GnRH signaling during the period of proportional growth. An acceleration of testicular growth was preceded by an increase in GnRH receptor mRNA levels and was accompanied by an increase in GnRH expression in the hypothalamus. Testis development was positively correlated with GnRH mRNA levels in the hypothalamus and was sensitive to stimulation by the GnRH agonist triptorelin and inhibition by GnRH antagonist cetrorelix during the period of accelerated growth. A possible mechanism of transition between “proportional” and “accelerated” periods at day 9 of rat life may be related to the developmental synchronization of GnRH neuronal activity generating GnRH pulses sufficient to affect testicular growth. This hypothesis needs further validation and investigation of the upstream and downstream mechanisms such as, for example, kisspeptine signaling [21] which may also be involved in acceleration of testicular growth during neonatal development.

In general, the acceleration of testicular growth in the 1.5-week-old rat and related neuroendocrine alterations for the first time described here may be useful for understanding mechanisms of neonatal human testis development that determine future male reproductive health [10], [57].
